# Advancing Oral Health Workforce Equity in Gender and Sexual Orientation

**DOI:** 10.1177/00220345251392066

**Published:** 2025-12-19

**Authors:** E. Ioannidou, M.W.B. Araujo, M. Bacino, C. Randall

**Affiliations:** 1 School of Dentistry, University of California San Francisco, San Francisco, CA, USA; 2 University at Buffalo, Buffalo, NY, USA; 3 School of Medicine, University of California San Francisco, San Francisco, CA, USA; 4 University of Washington, Seattle, WA, USA

**Keywords:** gender identity, sexual and gender minorities, gender equity, health care policy, workforce diversity, dental education

## Abstract

Sex, gender, and sexual orientation are multidimensional constructs that influence representation, inclusion, and outcomes in the oral health workforce. Despite demographic shifts in dental education, persistent inequities related to these identities remain underexamined in academic, research, and clinical settings. Our goal was to evaluate how sex, gender, and sexual orientation affect equity in the oral health workforce and to propose evidence-based strategies for advancing inclusivity in education and policy. We conducted a narrative synthesis using US-based data sources and peer-reviewed literature to assess demographic trends, historical structures, and institutional barriers. Particular attention was given to leadership disparities, economic inequities, and discrimination faced by women and LGBTQIA+ professionals (Lesbian, Gay, Bisexual, Transgender, Queer, Intersex, Asexual +). Best practices and policy strategies were also reviewed. Although women now constitute >50% of dental school enrollees, they remain underrepresented in leadership and surgical specialties and experience persistent wage gaps. LGBTQIA+ professionals face limited visibility, compounded bias, and a lack of structural protections. These disparities are amplified by intersecting identities, such as race and caregiving roles. Institutional gaps in inclusive curricula, faculty mentorship, and data collection further constrain equity. However, there have been some national initiatives that demonstrate promise for addressing these challenges. A more inclusive oral health workforce requires systemic reforms in academic policies, leadership development, cultural competency training, and federal financial support. Data collection on gender identity and sexual orientation, bias mitigation, and mentorship programs are critical levers for transformation. Aligning workforce policy with contemporary family structures and demographic realities can help ensure an equitable, representative, and resilient profession.

## Introduction

Sex, gender, and sexual orientation are often used interchangeably, yet they represent distinct intersecting aspects of human identity. *Sex* involves anatomic characteristics, physiologic traits including external genitalia, and secondary sex characteristics, as well as chromosomes and endogenous hormonal levels. *Gender* encompasses behaviors, roles, and identities constructed by society. *Sexual orientation* refers to patterns of emotional, romantic, or sexual attraction that determine how humans relate to each other (National Academies of Sciences, Engineering, and Medicine 2022). Understanding these 3 multidimensional constructs is essential for advancing equity within the oral health workforce, which has historically not determined the diversity of the populations that it serves, notably in terms of sex, gender, and sexual orientation.

As the dental profession evolves within increasingly diverse communities, it is imperative to develop a diverse and inclusive workforce to reflect those communities (Ioannidou and Bernstein 2021). Diversity in the dental workforce and in the health care workforce more broadly has been linked to improved access to care, enhanced patient trust and communication, and increased workforce satisfaction and retention (Stanford 2020; Ioannidou and Bernstein 2021). Yet, systemic barriers, including gender-based pay gaps, discriminatory environments, and a lack of representation, continue to affect those who do not identify with dominant norms (Okoro et al 2024). This article explores the role of sex, gender, and sexual orientation in shaping workforce equity within the oral health academic, research, and clinical environments. It draws on US-based data as one example of a developed country, with a particular focus on the implications for education and policy. In this article, we aim to examine historical and structural patterns, workforce demographics, barriers to inclusion, and the downstream effects on care and health equity, concluding with recommendations for a more representative and equitable oral health workforce.

## Historical and Structural Context

Dentistry and dental academia have long been dominated by men, particularly in leadership and surgical specialties (Istrate 2024). For much of the 20th century in the United States, <10% of practicing dentists were women (Tiwari et al 2019). Today, while women make up >50% of dental school enrollees, their representation in specialties such as oral surgery, academic leadership, and professional society governance remains disproportionately low. For example, because of the effect of traditional gender norms on the distribution of dental professionals across specialties, women more often enter pediatric dentistry or public health, which were more frequently and historically associated with caregiving. Meanwhile, men continued to dominate high-income and leadership-intensive fields such as oral maxillofacial surgery (Istrate 2024). Such historical context continues to shape the structure of the field today.

Historically and currently, the underrepresentation of LGBTQIA+ individuals  in dentistry is even more pronounced, and there is a paucity of data to indicate the true extent. Disclosure has been and remains risky in certain environments that lack explicit protections or inclusive policies and cultures (Sánchez et al 2015).

As such, only a few schools collect and/or report data on sexual orientation or gender identity, and this is a relatively new practice. For LGBTQIA+ providers, there are long-standing concerns about discrimination, patient bias, and professional repercussions—all of which contribute directly to a culture of silence and invisibility. Indeed, members of the LGBTQIA+ community are frequently overlooked, marginalized, and deprived of representation and often face disproportionate career obstacles, harassment, and professional discrimination as compared with their non-LGBTQIA+ colleagues (Westwood 2022).

 These historical trends and persisting structures reflect broader societal inequities, and they have tangible consequences: restricted access to mentorship, constrained career trajectories, and perpetuation of biased practices in care delivery (Smith and Norris 2025).

## Current Workforce Demographics and Trends

Based on data from the American Dental Education Association, the dental student population is now dominated by women, who represent >50% of the early-career dental workforce (Istrate 2024). However, this shift in entry-level roles is not yet reflected in higher levels of academic and clinical leadership, which continue to reflect the historical context more closely. The most current American Association of University Women (AAUW) report shows a persistent salary gap in the academic world, and additional evidence confirms that dental faculty women experience such salary gaps, followed by slower promotion rates, particularly after childbearing years (ie, the “motherhood penalty”; Ioannidou et al 2014, 2019; AAUW 2025). Leadership data further reveal gender disparities in leadership positions, where women held only 25% of dental dean positions across US institutions in 2023 (Istrate 2024).

In the United States, LGBTQIA+ people are 17% to 21% less represented in STEM fields (science, technology, engineering, and math) than expected. Oral health workforce–specific data regarding the representation of LGBTQIA+ professionals is largely unavailable, in part due to a lack of data collection and stigma-related fears. For instance, while some progress has been made in encouraging sexual orientation or gender identity disclosure in health profession education, most institutions still do not track or publish such data among faculty, students, or applicants. As a result, this invisibility maintains structural barriers and prevents truly inclusive environments.

These issues become more complex because the intersectionality of gender, sexual orientation, race, and class complicates workforce trends and policy requirements, as well as expectations. For example, BIPOC women (Black Indigenous, and People of Color) and LGBTQIA+ professionals face compounded marginalization, often navigating multiple layers of bias and exclusion in training and practice (Ioannidou and Bernstein 2021).

## Barriers and Disparities

Despite growing diversity in dental school student bodies, significant structural, institutional, and cultural barriers persist, affecting the recruitment, retention, and advancement of women and LGBTQIA+ oral health professionals.

*Economic disparities:* The AAUW (2025) highlights that women hold two-thirds of the nation’s student debt yet face chronic wage disparities throughout their careers. Even after controlling for specialty and years of experience, women in dentistry earn significantly less than their male counterparts (Ioannidou et al 2014, 2019; D’Souza et al 2019; US Census Bureau 2023). Therefore, the combination of greater student loan debt and smaller earnings puts women at an economic disadvantage that eventually is reflected in their retirement accounts (Bleiweis et al 2021).
*Leadership disparities:* Women remain underrepresented in senior academic roles, department chair positions, university presidents, and journal editorships. Specifically, white women represent 17% of all permanent presidents, while BIPOC women in aggregate compose <5% (Silbert et al 2022). This trend also applies to the representation of members of the LGBTQIA+ community in leadership positions at dental institutions (Tate and Glazzard 2025). Career interruptions for caregiving and a lack of mentorship often contribute to slower promotion. Consequently, the lack of representation in leadership limits women’s and LGBTQIA+ voices in decision-making processes affecting institutional policies and culture (Tate and Glazzard 2025).
*Work–life balance:* Considering social expectations, women are more likely to experience tension between professional and personal responsibilities, especially during early career and parenting years (Campus et al 2024). Few institutions offer formal support for part-time academic tracks, childcare, or caregiving leaves (AAUW 2025). In addition, the motherhood penalty is frequently observed in institutions where women tend to be pushed to part-time options and offered a pay cut because of parenting (Ishizuka 2021; Di Bartolo and Torres 2024; AAUW 2025).
*Discrimination:* LGBTQIA+ dental professionals report workplace microaggressions, exclusion from informal networks, and discomfort disclosing their identities. In states without explicit protections, fear of discrimination can be a barrier to employment and advancement (Fakhrjahani et al 2024; Sears et al 2024; Haley et al 2025; Smith and Norris 2025).

These barriers, which can be profound, affect not only individual careers but also the overall status of the profession, contributing to burnout, attrition, and reduced workforce diversity.

## Impact on Educational and Training Environments

Educational settings are central to shaping the future of the oral health workforce. However, many dental schools have not fully integrated equity-centered approaches into their curricula or faculty development, even though the integration of standards of care for all patients, including members of the LGBTQIA+ community, should be fully embraced when taking a humanistic approach to education and patient care (Commission on Dental Accreditation 2025).

*Curricular gaps:* Few predoctoral programs offer structured training on LGBTQIA+ oral health needs, gender-inclusive communication, or the intersection of social identity and oral health disparities. Inclusive content is often relegated to electives or optional workshops (Anderson et al 2009; Haley et al 2025).
*Faculty representation:* A lack of diverse faculty, especially those who are openly LGBTQIA+ or women of color, limits mentorship opportunities and impedes the creation of safe and inclusive learning environments (Tate and Glazzard 2025).
*Retention and promotion:* Biases in retention, evaluation, mentorship, and promotion processes continue to disadvantage faculty from underrepresented backgrounds. While pathway programs do exist, early career faculty still experience biases that exacerbate inequity and frequently lead them to exit the academic career path prematurely (D’Souza et al 2019; Ioannidou et al 2019).

Encouragingly, national initiatives are emerging. Programs such as the American Association for Dental, Oral, and Craniofacial Research’s MIND the Future, which supports early-career investigators from diverse backgrounds, are cultivating the next generation of leaders through mentorship and structured support. Still, these programs require greater visibility, funding, and institutional integration (Herren et al 2024).

## Patient Care and Public Health Implications

A diverse oral health workforce is not only a matter of fairness but is essential for improving individual patient care as well as public health outcomes. The workforce would need to mirror the general population. This allows patients to experience greater trust and satisfaction when providers share their identities or demonstrate cultural competence or cultural humility. Gender- and sexual orientation–concordant care can improve communication and health outcomes, especially in marginalized populations (National Institute of Dental and Craniofacial Research 2021). LGBTQIA+ individuals face higher rates of untreated oral disease, reduced access to dental care, and greater experiences of discrimination in clinical settings (National Institute of Dental and Craniofacial Research 2021; Fakhrjahani et al 2024). These disparities can be mitigated through inclusive provider education and workforce representation. Importantly, a workforce that reflects the diversity of its patient population promotes trust in the health care system and supports equitable care delivery. Moreover, inclusive practices and representation are especially critical in community clinics, public health dentistry, and educational settings.

## Strategies for Equity and Inclusion

Achieving a more equitable oral health workforce requires coordinated action across educational, institutional, and policy levels (Stanford 2020). Therefore, institutions should consider self-studies and subsequent intentional, impactful interventions. Effective self-studies should incorporate equity audits of promotion and tenure, compensation schedules, and mentoring programs for underrepresented faculty. In addition, hiring committees should promote appropriate and inclusive processes to ensure a large and diverse applicant group (Eckstrand et al 2017). To address academic attrition, institutional policies should align with contemporary family structures, including those with 2 working parents, single parents, and same-sex parents. As society has moved away from the “traditional family structure,” where one parent (usually the man) was the breadwinner and the other parent (usually the woman) was a stay-at-home caregiver, workforce policies have not readily synchronized with new structures (Miller 2020). Therefore, academic family policies need to consistently evolve to allow for flexible scheduling, paid parental leave, and affordable childcare programs.

To enhance workforce policy reform, extensive demographic data collection on gender identity and sexual orientation through anonymous surveys will provide a transparent and accurate picture of the oral health academic, research, and practice communities. Furthermore, educational programs could better integrate LGBTQIA+ health, structural competency, and cultural humility into core curricula (Gahagan and Subirana-Malaret 2028). They could also provide bias training and facilitate contemporary best practices for faculty and staff, especially those who are part of admissions committees and those who have supervision or leadership positions (Tamayo-Cabeza et al 2025). For instance, the implementation of an LGBTQIA competency course for dental students ranging from D1 to first-year postdoctoral residency showed that the course improved students’ understanding of health disparities faced by the LGBTQIA community, decreased biases felt by students prior to the course, and increased students’ confidence in treating the community (Salter et al 2024). While focusing on pathway programs, it is critical to support underrepresented minority students from their early stages of education and throughout all phases of professional and research training. In the meantime, in light of the current changes in federal policies in the United States and other countries, it will become important to reassess opportunities for financial support, scholarship programs, and loan forgiveness, especially for those trainees who go on to serve underserved communities. Based on the expectations, characteristics, and policy efforts stated here, outcome accountability could be measured and connected to state and federal funding as established in other countries (Donald et al 2011; Rosser et al 2019; Kalpazidou Schmidt et al 2020).

## Conclusion and Future Directions

The oral health profession stands at a pivotal moment. As societal understanding of sex, gender, and sexual orientation expands, so too must the systems that shape our workforce. Despite progress in representation, structural inequities continue to affect who enters the profession, who thrives within it, and who leads. There is a need to improve surveys and clinical research with specific assessments related to the LGBTQIA+ by using validated questionnaires that pay close attention to the needs and issues that affect the community.

This article has explored the role of sex, gender, and sexual orientation in shaping equity within the oral health workforce, with a focus on education and policy in the context of a developed country. The data reviewed here highlight persistent disparities in representation, pay, and leadership, especially for LGBTQIA+ individuals and women of color, and only marginally better patterns for other women as well as promising strategies for change.

Going forward, efforts must prioritize intersectional data collection, mentorship and leadership development, advocacy, and policy enforcement that holds institutions accountable to equity goals. Most important, efforts must foster an oral health workforce culture where every member regardless of identity can contribute, lead, and thrive.

## Author Contributions

E. Ioannidou, M.W.B. Araujo, C. Randall, contributed to conception and design, data acquisition, analysis, and interpretation, drafted and critically revised the manuscript; M. Bacino, contributed to conception, data acquisition, analysis, and interpretation, drafted and critically revised the manuscript. All authors gave final approval and agree to be accountable for all aspects of the work.
